# Use of a Smartphone App to Increase Physical Activity Levels in Insufficiently Active Adults: Feasibility Sequential Multiple Assignment Randomized Trial (SMART)

**DOI:** 10.2196/14322

**Published:** 2020-10-23

**Authors:** Bárbara De Barros Gonze, Ricardo Da Costa Padovani, Maria Do Socorro Simoes, Vinicius Lauria, Neli Leite Proença, Evandro Fornias Sperandio, Thatiane Lopes Valentim Di Paschoale Ostolin, Grace Angélica De Oliveira Gomes, Paula Costa Castro, Marcello Romiti, Antonio Gagliardi, Rodolfo Leite Arantes, Victor Zuniga Dourado

**Affiliations:** 1 Laboratory of Epidemiology and Human Movement (EPIMOV) Department of Human Movement Sciences Federal University of São Paulo Santos Brazil; 2 Department of Health, Education and Society Federal University of São Paulo Santos Brazil; 3 Department of Gerontology Federal University of São Carlos São Carlos Brazil; 4 Angiocorpore Institute of Cardiovascular Medicine Santos Brazil; 5 Lown Scholars in Cardiovascular Health Program Harvard TH Chan School of Public Health Boston, MA United States

**Keywords:** tailored messages, gamification, steps per day

## Abstract

**Background:**

The sequential multiple assignment randomized trial (SMART) design allows for changes in the intervention during the trial period. Despite its potential and feasibility for defining the best sequence of interventions, so far, it has not been utilized in a smartphone/gamified intervention for physical activity.

**Objective:**

We aimed to investigate the feasibility of the SMART design for assessing the effects of a smartphone app intervention to improve physical activity in adults. We also aimed to describe the participants’ perception regarding the protocol and the use of the app for physical activity qualitatively.

**Methods:**

We conducted a feasibility 24-week/two-stage SMART in which 18 insufficiently active participants (<10,000 steps/day) were first randomized to group 1 (smartphone app only), group 2 (smartphone app + tailored messages), and a control group (usual routine during the protocol). Participants were motivated to increase their step count by at least 2000 steps/day each week. Based on the 12-week intermediate outcome, responders continued the intervention and nonresponders were rerandomized to subsequent treatment, including a new group 3 (smartphone app + tailored messages + gamification) in which they were instructed to form groups to use several game elements available in the chosen app (Pacer). We considered responders as those with any positive slope in the linear relationship between weeks and steps per day at the end of the first stage of the intervention. We compared the accelerometer-based steps per day before and after the intervention, as well as the slopes of the app-based steps per day between the first and second stages of the intervention.

**Results:**

Twelve participants, including five controls, finished the intervention. We identified two responders in group 1. We did not observe relevant changes in the steps per day either throughout the intervention or compared with the control group. However, the rerandomization of five nonresponders led to a change in the slope of the steps per day (median −198 steps/day [IQR −279 to −103] to 20 steps/day [IQR −204 to 145]; *P*=.08). Finally, in three participants from group 2, we observed an increase in the number of steps per day up to the sixth week, followed by an inflection to baseline values or even lower (ie, a quadratic relationship). The qualitative analysis showed that participants’ reports could be classified into the following: (1) difficulty in managing the app and technology or problems with the device, (2) suitable response to the app, and (3) difficulties to achieve the goals.

**Conclusions:**

The SMART design was feasible and changed the behavior of steps per day after rerandomization. Rerandomization should be implemented earlier to take advantage of tailored messages. Additionally, difficulties with technology and realistic and individualized goals should be considered in interventions for physical activity using smartphones.

**Trial Registration:**

Brazilian Registry of Clinical Trials RBR-8xtc9c; http://www.ensaiosclinicos.gov.br/rg/RBR-8xtc9c/.

## Introduction

In Brazil, despite the economic crisis, the demand for smartphones has increased dramatically. There are approximately 324 million mobile devices connected to the internet in Brazil, of which 230 million are smartphones [1]. There is an enormous potential for smartphones to improve cardiovascular health in Brazil. Accordingly, efforts to engage people who do not meet physical activity recommendations have been made using popular emerging technologies, including mobile devices such as smartphones and their apps. There is evidence in the literature that app-based interventions to promote physical activity can be useful in yielding an overall moderate effect [2]. Regarding physical activity, a recent systematic review and meta-analysis reported that the use of wearables and smartphone apps led to a small to moderate increase in physical activity in minutes per day, and a moderate increase in daily step count [3]. However, recent evidence suggests that smartphone apps have been most effective in the short term (eg, up to 3 months), indicating the need for future research to establish the paths to improve physical activity in the long term [4].

In order to optimize app-based interventions for physical activity, a novel research design, namely the sequential multiple assignment randomized trial (SMART) design, might be a rational strategy. SMART is an adaptive design, which allows for alternative treatments depending on observed success in the intervention during the research period. This strategy brings the intervention more in line with real-life situations, helping to identify people who benefit from interventions differentially and individualize the treatment. Another benefit of this intervention design is the evaluation of multiple interventions and responses in one trial. In addition, the SMART design has been recommended over the classical randomized controlled trial for technology-based interventions [5,6], as it allows for adaptations over time based on the response to the intervention. This strategy may be beneficial, as the effectiveness of an app might diminish over time, because of losing interest in the app or its elements.

For example, consider a SMART to evaluate behavioral interventions in eHealth for scope and intensity. Assume that there are three types of strategies (A, B, and C), which are listed in order of dose and range. In this study, each participant would be randomized to one of two possible initial interventions (A or B). After a pre-established period, participants would be classified as nonresponders or responders, according to a previously defined criterion. Thereafter, nonresponders would be rerandomized to a subsequent intervention more rigorous in terms of the intensity and range of the initial intervention. Responders to treatments A and B would continue in their treatments to investigate the longer follow-up effect. Nonresponders to treatment A would be rerandomized to both receive treatment C and experience treatment B. Nonresponders in B, in turn, would be rerandomized to C or would change treatment by going to A. Six interventions are embedded in this design.

Given the need to evaluate the SMART design development and implementation process, as well as the preliminary results of each participant’s response to the proposed intervention, a feasibility study is appropriate and allows the identification of methodological aspects that may be adapted before more extensive randomized controlled trials. Moreover, conducting a pilot study favors the exploration of crucial outcomes beyond the evaluation of the study protocol implementation process [7].

Despite the high potential and feasibility [8] of a SMART design, so far it has hardly been utilized in smartphone and gamified interventions, especially for increasing physical activity. Accordingly, the primary objective of this study was to investigate the feasibility of a SMART design for assessing the effects of a community-based smartphone app intervention to improve physical activity in insufficiently active adults. We also aimed to describe the participants’ perceptions regarding the protocol and the use of the app for physical activity qualitatively. Moreover, we aimed to analyze the participants’ responses to the intervention.

## Methods

### Study Design

We conducted a feasibility study about the effectiveness of the SMART protocol using a smartphone app (free of charge) for the level of physical activity in adults. Our feasibility study expected to evaluate the recruitment capacity and resulting characteristics of sampling, evaluation, and refinement of data collection procedures; outcome measures; intervention and study acceptance and adequacy procedures; resources and ability to manage and implement the study and intervention; and preliminary responses to the intervention. The interdisciplinary team that developed the feasibility SMART study protocol included a psychologist, physical therapists, and professionals in physical education.

**Figure 1:**
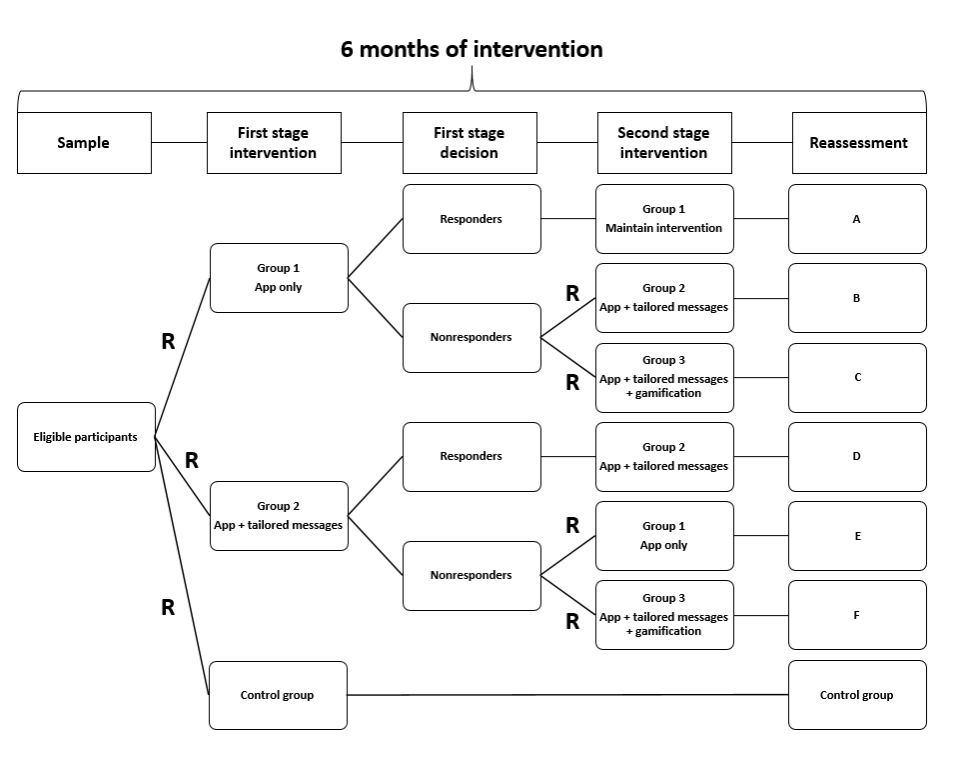
The sequential multiple assignment randomized trial (SMART) design applied in this study. Letters at the end of the flowchart indicate the way of the intervention. A: app only during 24 weeks; B: app only during 12 weeks and app + tailored messages in the last 12 weeks; C: app only during 12 weeks and app + tailored messages + gamification in the last 12 weeks; D: app + tailored messages during 24 weeks; E: app + tailored messages during 12 weeks and app only in the last 12 weeks; F: app + tailored messages during 12 weeks and app + tailored messages + gamification in the last 12 weeks; R: randomization and rerandomization.

This feasibility trial was a 24-week intervention with a two-stage SMART in which the allocation sequence of the randomization was concealed using opaque envelopes. An independent researcher performed this phase of the protocol. In the first stage of treatment decision, participants were randomized to group 1 (smartphone app only), group 2 (smartphone app + weekly tailored text messages), or a control group. After 12 weeks, based on the intermediate assessment (maintenance and increase or decrease in the number of steps), participants were classified as responders or nonresponders. The nonresponders were rerandomized into the two pre-existing groups (group 1 and group 2), and a new intervention group (group 3: smartphone app + weekly tailored text messages + gamification) was added to the protocol (Figure 1). Participants in the control group were advised to maintain their usual routine. The text messages sent to participants throughout the intervention are presented in Multimedia Appendix 1.

The ethics committee of the university approved this study (number: 0499/2018), and the trial was registered at the Brazilian Clinical Trials Registry (ReBEC #RBR-8xtc9c).

### Participants and Recruitment

As a rule of thumb, it has been recommended to recruit 30 participants for both pilot and feasibility studies, with samples between 24 and 50 being mathematically recommended to both calculate the standard deviation of a predetermined outcome and evaluate the rates of adherence and involvement (responder and nonresponder), as well as drop-out (for example) [9].

We invited 39 volunteers who presented to another ongoing study called the Epidemiology and Human Movement (EPIMOV) Study. Briefly, the EPIMOV Study is another study of our research team being carried out since 2013 in the city of Santos, Sao Paulo, Brazil. It is a prospective epidemiological study to investigate the association of physical activity and sedentary behavior with the incidence of cardiorespiratory and locomotor diseases. EPIMOV Study participants were recruited through social networks, folders displayed in the community, local magazines, and newspapers. In the EPIMOV Study, we included adults (age ≥18 years) who did not have cardiopulmonary diseases, locomotor disturbances, known electrocardiographic abnormalities, or other problems that would preclude them from safely performing physical exercises. EPIMOV Study exclusion criteria were regular use of assistive gait devices, recent respiratory infections, unstable or stable angina in the last 4 weeks, bradyarrhythmia or tachyarrhythmia, and abnormalities in lung function evaluated through spirometry. Thus, we used the participants of the EPIMOV Study in the sample of this SMART design study. All eligible participants, consecutively enrolled in the EPIMOV Study, were invited to participate in this feasibility trial, and upon agreeing to participate, they were randomized to one of the groups of the SMART design (Figure 1). In order to be eligible for the feasibility SMART study, the participants of the EPIMOV Study were required to be 30 years or older, be digitally engaged with their smartphones, and have a minimal behavioral change status (ie, higher than the precontemplative profile) for physical activity, based on the transtheoric model of behavior change at baseline [10].

The exclusion criteria for the feasibility SMART study were as follows: walking an average of ≥10,000 steps/day (assessed by a triaxial accelerometer) and/or a score of ≥3000 metabolic equivalents (METs)/min/week in the International Physical Activity Questionnaire (IPAQ) [11]. We set the limit of 10,000 steps/day since this is a well-recognized threshold for improving health [12] and more than 3000 METs/min/week in the IPAQ because it is related to the international recommendation for physical activity.

### Study Interventions

#### Tailoring Variables

We considered as the primary tailoring variable the increase in the average number of steps per day in comparison with baseline. Participants were rerandomized after 12 weeks from the beginning of the intervention, based on their response to the first stage of the intervention. In case of reaching the goal in the first stage, they remained in the same group; otherwise, they were exposed to a new intervention (Figure 1). The participants were informed that they would be joining an adaptive trial and that there was potential for rerandomization if they did not respond to or use their first intervention condition.

We asked participants to increase their average daily step count by 2000 steps as much as possible. There is no consensus about the minimum increase in the number of steps (per day or per week) related to cardiovascular health improvement, although one study found that a change by 2000 steps/day was inversely associated with the risk of a cardiovascular event [13]. Moreover, the American College of Sports Medicine recommendations on the quantity and intensity of physical activity suggests an increase of an average of 2000 steps/day for benefits in cardiovascular health [14].

**Figure 2:**
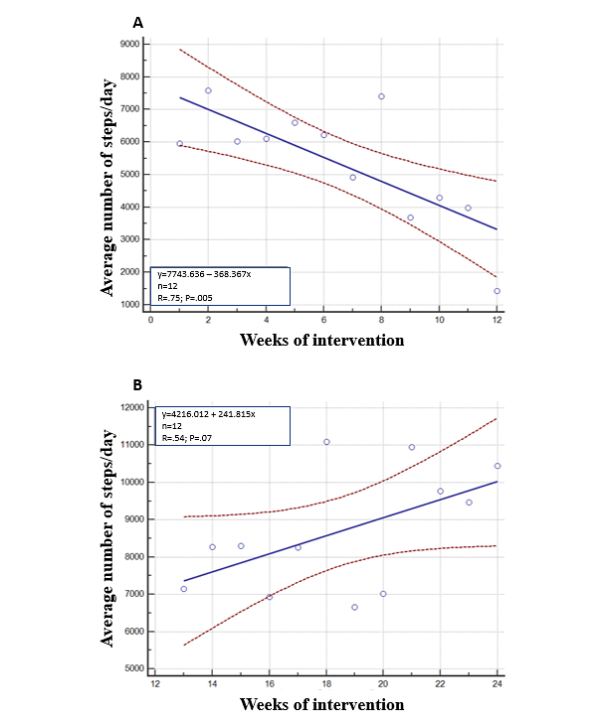
The method developed in this study to define responders and nonresponders to the smartphone app intervention for physical activity. (A) An unresponsive participant in the smartphone app only intervention; (B) The same participant after rerandomization to an intervention combining the app with gamification features.

However, at the very beginning of the intervention (third week of the sixth participant assessment), we decided not to use the increase of 2000 steps/day for identifying responders and nonresponders in order to avoid making participants feel discouraged or uncomfortable by reaching the goal only a few times. Thus, the goal for each participant was any increase in the daily step count compared with the initial assessment. In this way, to define responders and nonresponders in this study, we fitted a linear regression for each participant with the relationship between weeks on the x-axis and the number of steps per day on the y-axis (Figure 2). Thereafter, we considered responders as those with any positive slope at the end of the 12-week first stage of the intervention. Those with zero or negative slopes were considered as nonresponders.

##### Group 1

In group 1 (smartphone app only), participants were instructed to monitor their daily steps using the Pacer app. Additional instructions on app features were not provided, and encouraging text messages were not sent. In order to record the average number of steps per week, initially, participants were instructed to send a print screen of their step counts every Monday. After it was possible, we began to collect this information using the app interface by verifying each participant profile on the app with their agreement. Participants also received weekly questionnaires with questions, such as how they felt about that week, what was the appropriateness of the goal imposed on them, and how much they would like to remain in the study.

##### Group 2

In group 2 (smartphone app + weekly tailored messages), participants used the app to track the number of daily steps, and they received weekly text messages on their smartphones with information about their performance in the previous week and motivational messages according to their behavior change status [15]. We prepared a series of 48 messages (24 directed to participants with a contemplative behavior change profile and 24 directed to those with preparation and action behavior change profiles). The 24 messages included 12 for those who reached the goal and 12 for those who did not reach the goal. We sent the messages on the same weekday using a free app. Furthermore, the weekly questionnaires were sent in the same way as in group 1.

##### Control Group

Participants in the control group were instructed to maintain their usual routine.

##### Group 3

In group 3 (smartphone app + weekly tailored text messages + gamification), in addition to app use and tailored text messages, participants were instructed to form groups with researchers to use the functions available in the app as described above. The researchers in this group acted both as dummy participants and as social moderators, competing and giving encouragement to the real participants. Apart from step monitoring and individual messages, participants were encouraged to join virtual challenges. For each challenge they completed, they were rewarded with a virtual badge on the app. Challenges available were as follows: target number of steps per day, target distance walked in the month, and group competitions where the total number of steps was compared among different groups of app users. In addition, there were rankings of the number of steps among all users, as well as running challenges.

At the end of the sixth month, there were seven possible ways of the intervention. These ways were identified as the way in the control group (one way) and ways with the letters A to F (six different ways) as follows: A, app only during 24 weeks; B, app only during 12 weeks and app + tailored messages in the last 12 weeks; C, app only during 12 weeks and app + tailored messages + gamification in the last 12 weeks; D, app + tailored messages during 24 weeks; E, app + tailored messages during 12 weeks and app only in the last 12 weeks; F, app + tailored messages during 12 weeks and app + tailored messages + gamification in the last 12 weeks (Figure 1).

#### Outcomes and Assessments

Study outcomes were assessed at baseline and after 12 and 24 weeks by researchers blinded to group allocation. All researchers who did the assessments were blinded. The researchers who did the group allocation, sent messages, and participated in gamification were not blinded. At each scheduled assessment period, study measurements were carried out during two visits, spaced 7 days apart. In all three assessments, participants repeated the protocol of the two visits. In the first visit, participants underwent general health screening (clinical and sociodemographic characteristics), assessment of the behavior change status for physical activity [15], anthropometric assessment, lung function assessment, and cardiorespiratory fitness assessment. At the end of the first assessment, participants were informed about using the triaxial accelerometer for the subsequent 7 days for the assessment of the average number of steps per day. At the end of the first visit, they were also instructed to install a smartphone app for physical activity monitoring and to use it throughout the subsequent 7 days, so that we could establish a personalized goal regarding the increase in steps per day. In the second visit, they returned the accelerometer, and we assessed the physical activity levels and body composition.

#### Clinical and Sociodemographic Assessments

Baseline assessments included the age, sex, race, and educational level of the participants. We measured height (m) and body mass (kg) in all participants. Thereafter, we calculated the BMI and defined obesity as a BMI30 kg/m^2^. We also investigated by self-report the presence of previous diagnoses of the main risk factors for cardiovascular disease, including systemic arterial hypertension, diabetes/hyperglycemia, and dyslipidemia/hypercholesterolemia. A family history of premature coronary heart disease was defined as myocardial infarction or sudden death before 55 years of age in the father or another male first-degree relative, or before 65 years of age in the mother or other female first-degree relatives. We also asked participants about current smoking.

#### Physical Activity Behavior Change Status

We assessed participants’ behavior change status for physical activity according to a previously validated questionnaire [15]. This questionnaire provides information about the physical activity habits of the volunteers and the plans to start a physical activity behavior, which was used to develop personalized messages, as will be described later.

#### Sedentary Behavior and Physical Activity Levels

We performed this evaluation with a validated triaxial accelerometer (ActiGraph GT3X+, MTI) [16]. Participants wore the device for 7 consecutive days of assessment during the waking hours. To be considered valid, days of data collection needed to have at least 10 hours of continuous monitoring, starting at the moment of awakening. Participants used the accelerometer until bedtime, except during showering and aquatic activities. Nonwearing time and the thresholds for the intensity of physical activity were evaluated as previously described [17]. We defined wearing time as 24 hours minus nonwearing time. Periods of zero counts for 60 or more consecutive minutes were considered as nonwearing time. To be considered as valid data for analysis, volunteers needed to use the device for at least 4 days (10 hours/day), including a weekend day.

The total amount of sedentary behavior was considered based on the minutes with less than 100 counts/minute (cpm), which represents <1.5 METs of energy expenditure. We evaluated sedentary behavior, light-intensity physical activity, and moderate to vigorous physical activity at baseline and at the end of the 24-week intervention. The measurements were calculated in hours per day considering the total wear time and the number of calendar days of use, as well as in percentage of the total time. The thresholds for the intensity of physical activity were as follows [17]: (1) light physical activity (100-1951 cpm) and (2) moderate to vigorous physical activity (>1951 cpm). Physically inactive participants were considered as those participants with less than 150 minutes/week of moderate to vigorous physical activity or less than 75 minutes/week of vigorous physical activity [14].

#### Daily Step Count and Smartphone App

We obtained the baseline average daily step count by using a smartphone app. Before starting the intervention, we tested several smartphone apps. Researchers installed on their smartphones the most popular free physical activity apps with a step-monitoring function that worked correctly in both Android and iOS operating systems. After a meeting, we decided to use the Pacer app. We agreed on this choice based on some features of this app. First, it handles well on the two most popular operating systems. Second, it accurately monitors daily steps. Third, it has gamification features, such as goal setting, rewards, virtual badges, progress bars, walking/running rankings, and group formation possibility. Finally, the app has social network and coaching functions.

### Data Analysis

#### Qualitative Data Analysis

We performed qualitative analysis of the perceptions of all participants included in the SMART in terms of the protocol and the use of the app for physical activity. The exchange of text messages with participants was performed through the free instant messaging app WhatsApp. We transcribed to text all the text messages exchanged between participants and researchers. The proceedings used were adapted from qualitative research in health [18]. To assimilate the content of the material, we conducted a free-floating reading of the transcribed material, followed by an exhaustive reading until recording units were extracted. Finally, we organized and analyzed the material according to the literature recommendations.

Conversations with participants via the app occurred two times a week and were usually initiated by the researcher. The content of these conversations started with an initial greeting, talked about the participant’s inclusion, and included instructions on using the step counter app and instructions on sending a print screen with weekly steps. Conversations also involved reports on performance of the participants, health situations, and difficulties in handling the app, where assistance was provided. Participants could answer the messages sent by researchers through text and emojis (message app feature).

#### Quantitative Data Analysis

Quantitative analysis involved comparison between the slopes of the average number of steps per day obtained in the first stage of the intervention (12 weeks) and the second stage (24 weeks). Because of the small sample size, we chose the following statistical procedures. We performed a Mann-Whitney test to investigate the differences in the study groups. We compared the average number of steps per day at baseline and after 12 and 24 weeks of the intervention using the Kruskal-Wallis test. We also used an independent samples *t* test for comparisons between intervention groups and the control group at baseline and at the end of the 24-week intervention. Finally, we fit linear and quadratic regressions both during the first 12 weeks of the intervention and overall to investigate the behavior of changes in the average number of steps per day throughout the intervention. As for the linear trend in the number of steps, we calculated the slopes for each participant who finished the protocol (stages one and two) and then compared the median of the slopes using the Mann-Whitney test. Moreover, we calculated the median of the weekly number of steps of the five participants who completed the protocol and compared the slopes of the step trends between the initial 12 weeks and the final 12 weeks using analysis of covariance. We used the number of steps as the dependent variable, the intervention stage as a fixed factor, and the intervention weeks as a covariate. We set the alpha level at 5% for all analyses.

## Results

**Figure 3:**
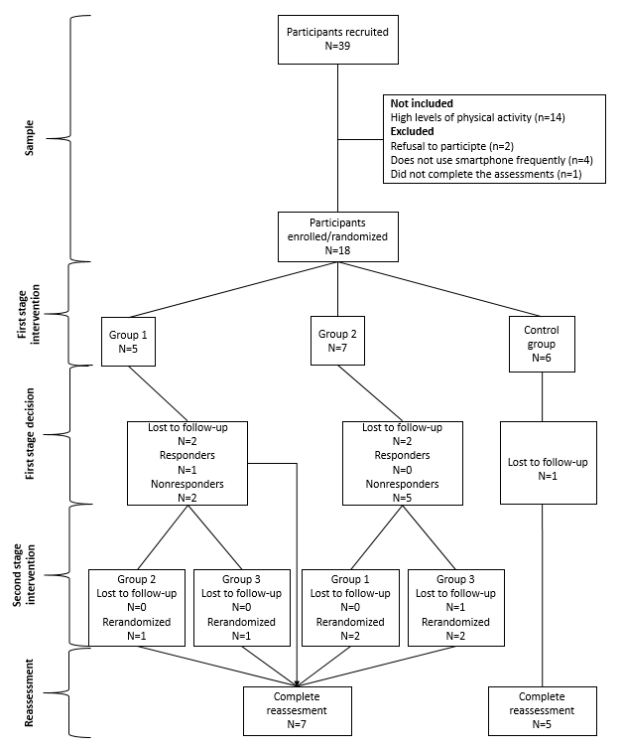
Flow chart of the feasibility sequential multiple assignment randomized trial (SMART) utilized in this study.

In total, we invited 39 participants from the EPIMOV Study. Among them, 14 were not included (presented a high physical activity level, ie, >10,000 steps/day or >3000 METs in the IPAQ) and seven were excluded (two refused to participate, four reported not using a smartphone in daily life, and one did not complete the assessments) (Figure 3). Of the 18 participants randomized, seven in the intervention groups and five in the control group finished the 24-week intervention protocol (Figure 3). We found no relevant differences between groups regarding the general characteristics at baseline (Table 1).

**Table 1:** General characteristics of the study participants.

Characteristic		Intervention groups^a^	Control group^a^	*P*
Age (years)		44 (7)	42 (7)	.64
Sex (male/female)		4/3	4/1	.54
Weight (kg)		90 (27)	89 (25)	.95
Height (m)		1.69 (0.11)	1.63 (0.12)	.39
BMI (kg/m^2^)		31.3 (9.0)	33.2 (8.9)	.71
**Physical activity**				
	Sedentary (h/week)	55.2 (12.4)	55.7 (12.6)	.68
	Light intensity (h/week)	19.9 (5.8)	24.7 (6.6)	.28
	Moderate to vigorous intensity (h/week)	2.95 (1.19)	2.55 (1.54)	.62
	Average number of steps/day	3910 (2097)	4170 (2024)	.27

^a^Data are presented as mean (SD) or n/n.

We successfully adopted the SMART design, performed randomization and rerandomization among all groups, and delivered the proposed intervention for a feasibility study. The technology-based intervention allied with the SMART design may have created conditions to favor participants’ behavior change. The delivery of tailored messages, identification and rerandomization of nonresponders, and interaction between participants and researchers were feasible. The average number of daily steps was a feasible measure of the level of physical activity for this study design.

The qualitative analysis showed that participants’ reports could be classified into the following three categories: (1) difficulties in managing the app and technology or problems with the device, (2) good responses to the app, and (3) difficulties in achieving the goals (Table 2). An example of a participant-researcher interaction is shown in Textbox 1.

**Table 2:** Qualitative data results.

Category	Participants’ quotes
(1) Difficulties in managing the app and technology or problems with the device	*I think there’s some problem. It’s zero for some days. Back to normal.* [Participant #01, male, 37 years old] *I changed the phone and could not use the app. Do you know how could I restore the data? I tried but couldn’t do it.* [Participant #14, female, 37 years old] *But the data won’t be correct because the app hasn’t been working well, that’s why I don´t have it with me all the time. When I go out for a walk, it does not work, and at home, it sometimes does. Also, there are some clothes it doesn’t fit in the pocket… got it?* [Participant #11, female, 52 years old]
(2) Good responses to the app	*This week I’m doing well, I’ve been hiking and running 6k.* [Participant #04, female, 33 years old] *I have a friend who would like to [participate], is there any chance?* [Participant #07, female, 62 years old] *Still trying to commit me to the goals and my work schedule and the knees...* [Participant #09, female, 47 years old]
(3) Difficulties in achieving the goals	*Good morning! I know I have to improve and also that it is a shame these steps, but it’s not because I want to, unfortunately, if it’s not one thing it’s another but I’ll try.* [Participant #04, female, 33 years old] *Good afternoon. Rainy week. Cut me some slack. Hugs.* [Participant #06, male, 51 years old] *I didn’t reach the proposed goal, and health is so-so.* [Participant #09, female, 47 years old] *And I’ve been very busy with my orders, thank you and have a great day.* [Participant #11, female, 52 years old]. *Vacation last week.* [Participant #14, female, 37 years old]

**Example of participant-researcher interaction messages.**

Researcher: Good morning! Your average number of steps this week was 8,281/8,208.

Participant #14 (female, 37 years old): Good morning.

Researcher: [emoji reinforcing the participant’s behavior]

Participant #14: [emoji expressing happiness]

Participant #14: I started a bodybuilding program.

Researcher: Cool! Congratulations!

Only one out of 12 participants achieved the goal of increasing 2000 steps/day in the first 12 weeks of the intervention and presented a positive slope. Therefore, the participant was classified as a responder and was not rerandomized. Among nonresponders, one out of seven participants achieved the goal in the remaining 12 weeks after rerandomization. On occasions when the goal was not reached, in 66.7% of responses, participants reported feeling bad or very bad about it. Interestingly, in 72% of weekly questions, they reported that the 2000-step goal was adequate at the time they did not achieve it. In addition, in 99% of cases, participants reported being willing to continue the study. In 95% of cases, participants reported feeling well or very well when they reached the goal of increasing 2000 steps/week. They also reported performing physical activities alone 66% of the time. Walking for exercise and leisure were the most prevalent types of physical activity.

**Figure 4:**
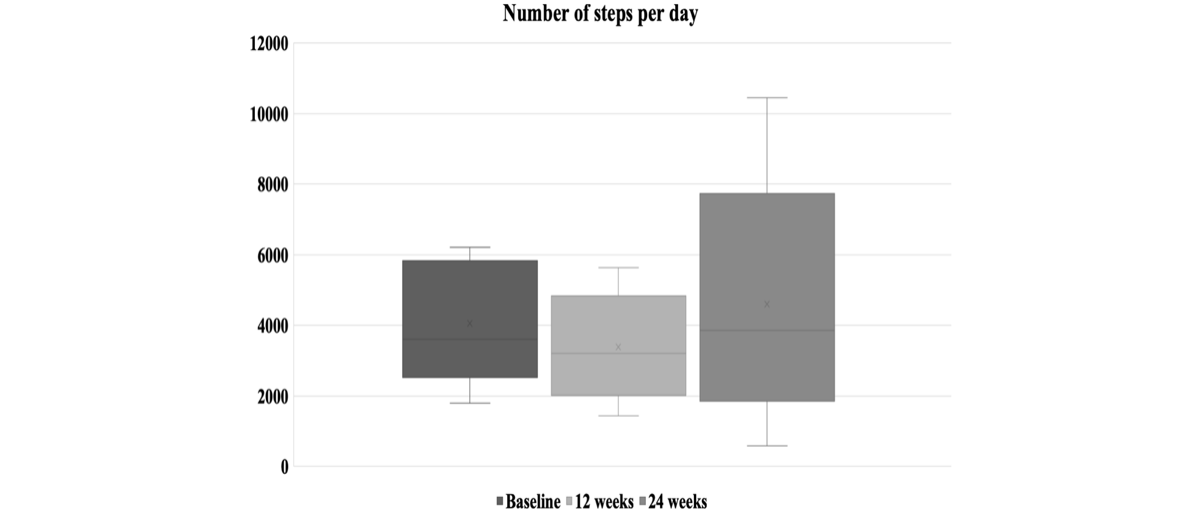
The average number of steps per day at baseline, after 12 weeks of the intervention, and after rerandomization and new intervention adoption up to 24 weeks of the study protocol.

**Figure 5:**
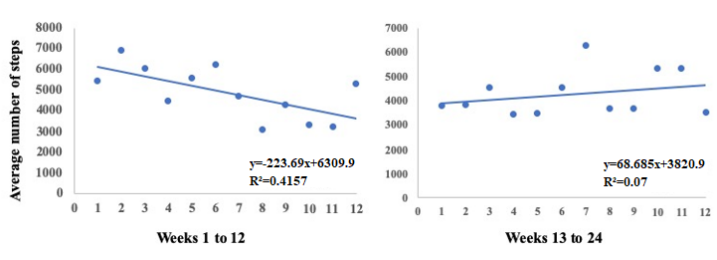
Linear regressions with the slopes of the relationships between the median number of steps per day of the five participants who finished the protocol and weeks of the intervention. The slope of the second stage of the intervention (weeks 13 to 24) was significantly different compared with the first stage of the intervention (*P*=.02).

**Figure 6:**
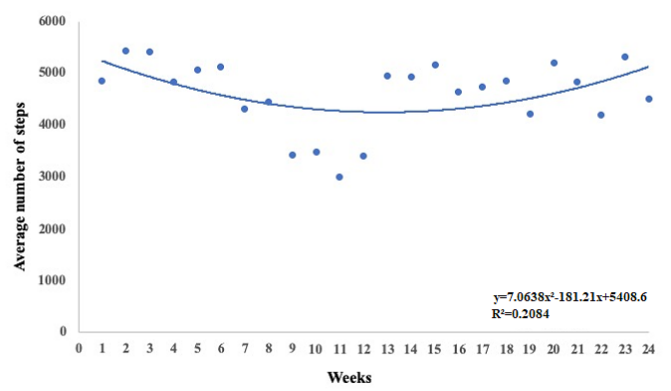
The average number of steps per day throughout the 24-week intervention showing a quadratic relationship in the total sample of participants who finished the protocol.

We did not observe significant changes (*P*=.10) in the average number of steps per day on comparing baseline, week 12, and week 24 (Figure 4). Moreover, we found no differences in the average number of steps per day among groups at baseline (Table 1) and at the end of 24 weeks of the intervention (intervention groups: mean 3995, SD 3204 vs control group: mean 4250, SD 4204). However, we observed that after rerandomization (week 12), participants tended to change the slope of the average number of steps per day (from median −198 steps/day [IQR −279 to −103] to median 20 steps/day [IQR −204 to 145]; *P*=.08). Comparing the trend of the median number of steps between the first and second stages of the intervention in the total sample, we found a relevant inflection (Figure 5). Considering all 24 weeks of the intervention, we observed that four out of five nonresponders presented a quadratic relationship between the average number of steps per day, with positive inflection after rerandomization (Figure 6). We observed these results regardless of the sequence of interventions.

As for responders, we identified one participant with a slope showing an increase of 8 steps/week in the first stage of the intervention, who maintained a positive slope of 26 steps/week in the second stage of the intervention. Another participant in group 1 showed an increase in the number of steps with a slope of 1341 steps up to the sixth week of the intervention before dropping out of the study.

**Figure 7:**
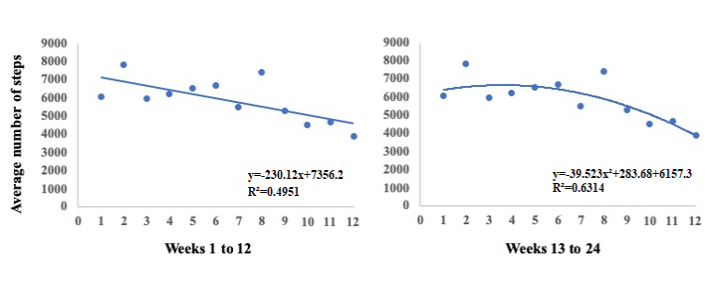
Examples of better fit using quadratic regression (A) compared with linear regression (B) for predicting the behavior of weekly changes in the average number of steps per day.

Finally, we observed that for three nonresponders from group 2 (smartphone app + tailored messages), a quadratic rather than a linear regression was better to predict the behavior of the average number of steps during the first 12 weeks of the intervention (Figure 7). They showed an increase in the number of steps up to the sixth week and then presented an inflection to baseline values or even lower.

## Discussion

### Principal Findings

The primary objective of this study was to investigate the feasibility of a SMART design for assessing the effects of a community-based smartphone app intervention to improve physical activity in insufficiently active adults. We also described the participants’ perceptions regarding the protocol qualitatively. Moreover, we aimed to analyze the participants’ responses to the intervention. Supporting the primary purpose, we observed that this study design was feasible for interventions promoting behavior change in physical activity. Furthermore, multicomponent app-based interventions seem to be more effective than app-based interventions alone [19].

We showed that the SMART design, in association with behavior change techniques and technology-based features, is feasible and shows potential to be effective in promoting more active lifestyles. The study design worked correctly and called for participants to experience positive effects on physical activity levels. Thus, the SMART protocol required adequate planning and a dedicated interdisciplinary team to deliver the intervention. Interdisciplinary interventions increase the chances of achieving the different dimensions of change and sustenance of complex behaviors, as in physical activity [20].

After we finished this feasibility SMART study, we made changes to the protocol, and the full trial is ongoing. For the adaptive intervention trial itself, we will change the way we recruit volunteers. Initially, we were inviting participants from another study with a different focus (EPIMOV Study) to be part of the SMART study. However, we realized that we need to broadly publicize the SMART study (social media, networks, and newspapers) to recruit volunteers interested in increasing their levels of daily physical activity, which we hope will contribute to decreasing the drop-out rate.

The qualitative analyses showed that participants presented an excellent response to the app, as well as important issues such as difficulty in managing the technology and difficulties in achieving the goals, which could be addressed in a future large-scale clinical trial. The possibility of interaction with the team had an essential role among participants. It is interesting to observe that participants tried to report their difficulties or their achievements/progress to the researcher. The sensibility and acceptance of the participants’ demands had an essential role in the process of behavior change among the participants of this study. Some people need more support and incentive to start the change, and some recent evidence showed that self-efficacy is a potent mediator for improving physical activity, especially considering meaning in life and peer support [21,22]. The researcher-participant interaction reinforces the positive effect for the participant trying to change behavior.

Researchers in the field of psychology point out the complexity and importance of therapeutic alliance research in psychotherapy [23,24]. Therefore, broadening the understanding of such variables in intervention programs that seek to change behavior is relevant. Although it is a topic that needs to be better understood, we believe that considering the importance of the quality of the relationship established between the researcher and participant is a crucial aspect in the process of change of human behavior.

Smartphone devices and apps provide more awareness than motivation in practicing physical activity [25]. The interaction of a participant with a professional evaluator in physical activity and health brings higher reliability and promotes the overcoming of physical barriers or geographic isolation. It has been shown to increase adherence and involvement in physical activity intervention programs, overcome barriers, and increase motivation to achieve goals [25]. This intervention proved to be effective in a 10-week physical training program submitted through WhatsApp, along with sending encouraging messages and responses to any questions from participants. Muntaner-Mas et al proved that it was feasible and had good adherence, even without the use of behavior change techniques, although the researchers emphasized the importance of such techniques [26]. This result is in line with our study design, in which we performed a SMART owing to the adaptive characteristic of this design.

Furthermore, the availability of researchers allied with the weekly text messages may have contributed to participants’ adhesion to the intervention since they could stop using the app or stop monitoring or trying to increase their daily steps. Although electronic devices and apps are essential tools for health interventions, real-time feedback is crucial for behavior change [27].

In a small sample, we found a tendency to increase the average number of steps per day throughout 24 weeks of the intervention, especially in the last 12 weeks. Moreover, the tailored messages seemed to have a positive impact on the physical activity level; however, our results demonstrated that rerandomization to a new treatment strategy would be better implemented before 12 weeks of the intervention. These findings confirm previous research about the importance of the social environment [28,29] and the use of messages as strategy motivation to increase the physical activity level [30]. Given that levels of motivation may vary across the lifespan [31], a combination of strategies to keep individuals physically active seems to be more effective [32]. Studies have shown that the social environment has an essential influence on the psychological and behavioral aspects related to the level of physical activity [33].

Our findings suggest that the rerandomization itself seemed to play an important role in participants’ behavior, leading to an inversion of the tendency to decrease physical activity over a short period. While most participants were not able to achieve the goal of increasing 2000 steps/day, we observed, intriguingly, that they reported that this goal was adequate.

After about 3 weeks of the intervention, we decided to redefine the goal to avoid making participants feel discouraged or uncomfortable by reaching the goal only a few times. The importance of goal setting has been discussed in the field of sports psychology. Weinberg [34] argues that, for setting a goal, an important principle is that it should be challenging and realistic. If goals are too complicated, the tendency is that individuals lose motivation and give up, and if it is too easy and does not present a challenge, individuals become complacent and do not reach maximum effort [34].

Thus, the goal for each participant was to increase their daily steps by any count compared with the initial assessment. This goal can be an essential strategy to encourage self-regulation, which plays a vital role in behavior change. Buckley et al [35] showed that cognitive control abilities play an important role in the self-regulation of physical activity and sedentary behavior. Self-regulation may be defined as a process that permits an individual to guide his activities over time and circumstances. It consists of the modulation of thoughts, attention, affects, or behavior by deliberate or automated use of cognition [36].

In addition, we consider that the presentation of personalized messages sensitive to the stage of behavior change can generate a higher positive response and incentive in the search for a goal. In the initial stage of behavior change, the possibility of the individual perceiving himself/herself as supported and encouraged is of fundamental importance for the construction of a more effective behavior change [37]. Sending automated messages produced a null effect on increasing physical activity in patients with type 2 diabetes [38].

Finally, our results showed a decline in the average number of steps of participants in the first stage of the intervention with relevant positive inflection in the second stage. However, it is worth noting that an essential part of the sample that received personalized messages showed quadratic behavior in the step trend with an evident decline from the sixth week. These results suggest that if rerandomization was performed earlier, our results could have been even more consistent. Our intervention currently provides for rerandomization in the sixth week. Adaptations in technology intervention are dynamic and must be implemented quickly. In this sense, an application with sufficient artificial intelligence could automate and individualize the adaptive process to increase physical activity.

### Strengths and Limitations

As a strength of our study, we highlight its novelty. To our knowledge, this is the first study to develop an adaptive intervention based on behavior change techniques to increase the level of physical activity in adults. In addition, an interdisciplinary team was mandatory for the proposed intervention (ie, behavior change for physical activity). We showed that the SMART design, in association with behavior change techniques and technology-based features, is feasible and shows potential to be effective in promoting a more active lifestyle. In addition, few participants reported problems using the app. The Pacer app was useful for step counting, as expected. Even with limited accuracy, the number of steps taken in the Pacer app was reproducible and able to predict the distance traveled during walking [39]. Therefore, continuous monitoring of the number of steps through the app in this study was adequate. We were able to conduct the SMART design study, which required adequate planning and a dedicated team to deliver the intervention. The study design worked correctly and called for participants to have positive effects on the physical activity level. Finally, the qualitative analysis provided relevant information that may be useful to plan interventions on physical activity behavior change.

We are aware that the 34% drop-out rate of participants in this study is a possible limitation. However, this proportion has been described in other physical activity interventions in primary health care, even with a shorter intervention period (eg, 12 months) [39-41]. Moreover, we recognize the small number of participants, which limits pre- and postintervention comparisons, but this was a feasibility study, and we worked on improvements before beginning the full trial. Finally, we had no information about more specific feedback from participants on how they felt about achieving the increase in the physical activity level proposed. However, we intend to improve this point by forming focus groups with participants who go through the design of the study.

### Conclusion

The SMART design was feasible for assessing the effects of a community-based smartphone app intervention to improve physical activity in insufficiently active adults. Our results suggest that rerandomization should be implemented earlier to take advantage of tailored messages.

Additionally, difficulties with technology and a realistic and individualized goal should be considered in interventions for physical activity using smartphones. We found a tendency to increase the average number of steps per day throughout the 24 weeks of the intervention, especially in the last 12 weeks. The results from the feasibility study contributed greatly to the final design of the SMART.

## Appendix

Multimedia Appendix 1 Text messages sent to participants throughout the intervention.

## Abbreviations

cpm: counts per minute
EPIMOV: Epidemiology and Human Movement Study
IPAQ: International Physical Activity Questionnaire
MET: metabolic equivalent
SMART: sequential multiple assignment randomized trial
