# Supplementary material for: Use of a Smartphone App to Increase Physical Activity Levels in Insufficiently Active Adults: Feasibility Sequential Multiple Assignment Randomized Trial (SMART)
Source: JMIR Res Protoc. 2020 Oct 23;9(10):e14322. doi: 10.2196/14322 (PMC7647811; doi:10.2196/14322)
Supplement: Multimedia Appendix 1 [file resprot_v9i10e14322_app1.docx]

**NEGATIVE/NOT ATTACKED THE GOAL - PCCN (pre-contemplative and contemplative NEGATIVE)**

PCCN 01- How are you? All the effort is rewarded, and your health thanks you. How about this week trying to go up and down as many steps as possible? It would be a significant challenge. I know this week was harder to reach the goal, but stay firm. We are happy with your results so far. Do not be discouraged.

PCCN 02- We realized this week it was not possible to reach its goal. Becoming more active leads to a healthy life; increasing the number of steps also improves life quality. We are together with you in this goal, do not give up! Thank you for your daily dedication.

PCCN 03- Hi, how are you? Sometimes it seems complicated to achieve goals that we set in our lives, but this becomes possible gradually with persistence. Congratulations on your efforts. You have shown a lot of power in this first phase. Remember that each step further is a positive point for the path that leads to a healthy life. Keep focus!

PCCN 04- How is it going? Our goals are attainable and prepared for you, so we trust that you will reach out to it. Thank you very much for your commitment to the project. We have seen that you are striving to achieve the results, and your health is grateful. How about a walk to a close friend to chat? It will be a good and pleasant time.

PCCN 05- Hello, we are following your evolution and seeing that the active life is increasingly present in your daily life, but this week we can not reach the stipulated goal. How about this week that begins to walk through streets that you usually do not transit? In addition to promoting health would be a great way to meet new places. Congratulations on your commitment.

PCCN 06- How are you? Changes in habits sometimes seem complicated. Do not be afraid of change. It sometimes scares you, but it can be the key to that door you both crave open. Come on! You should increase your number of daily steps. Change the habit.

PCCN 07- Hi, how are you? We are following your evolution, and we have seen this week that you have been less active. Faced with difficulty, replace the "I can not" with the "I'll try again." In the battle against sedentarism, only those who fight will win. We're here to help you.

PCCN 08- Hello, we can not reach the goal this week. Do not give up. Difficult is not to fight for what you want; the challenge is to give up what you love. Love your body. Take care of your health. We are together with you in this fight. How about a walk?

PCCN 09- How's everything going? This week was harder for us to meet our goal. Remember that the cycle of life is to try, fall, rise, and start again, without ever giving up. Let's move on. We hope your goal will soon be part of your daily habits. Let's try to change just one habit of your day to day, to soften our goal.

PCCN 10- Hi. We are following how hard you have been working towards your goal, but this week we still cannot. Think you may even not get where you wanted to go, but indeed you are closer to your goal today than yesterday. How about a night walk on the beach to put your thoughts in order, admiring a beautiful landscape?

PCCN 11 - How are things okay? Today we find that you have failed to reach your goal. There is much talk today about "detachment." How about getting into fashion and "detaching" from a sedentary life to get a healthier life? We're sure you can achieve your goal this coming week. We trust you.

PCCN 12- Hello, we are following your evolution, and seeing that the active life is increasingly present in your daily life, but this week, we cannot reach the stipulated goal. Sometimes it is difficult to make time for physical activity in our routine, but a healthier life, avoid that we have to take time to take care of lost health in the future! How about this week increasing the number of steps on the way to a healthy life? We count on you!

**NEGATIVE/NOT ATTACKED THE GOAL - PAMN (preparation, action, and maintenance NEGATIVE)**

PAMN 01 - Hello! We know you're focused on healthy living, that's great! But we evaluated this week that we were not able to reach our goal. How about a challenge this week? Walk two more blocks after work. Do you accept the challenge? We count on you!

PAMN 02 - How's it going? We realized this week that you had difficulties in achieving our goal. I think this weekend, a walk on the beach would be perfect for your physical and mental health. From channel 6 to channel one, we have 4,235 meters, it will be a great challenge, and your heart is grateful. Congratulations on the engagement.

PAMN 03 - Hi. Lifestyle changes are always challenging. It is common to discourage at first, but we have to maintain perseverance to achieve the goals. Did you know that the average distance between one channel and another in Santos is almost 1 km? When we walk on the beach, the view is so good that we do not physically feel the distance traveled. Let's stay focused! How about a walk along the waterfront this week?

PAMN 04 - How's it going? Your number of steps shows that you have been dedicating. However, this week reaching the goal was not possible. We know that sometimes new challenges are difficult to achieve, but do not be discouraged. Try to less and less use the car to move to places. Thank you very much for your participation. Hug.

PAMN 05 - Hi! You can stay focused and have been changing your lifestyle for the better, each day, with your involvement in increasing the number of daily steps. With this, you decrease with each step, the risk of diseases related to the sedentary lifestyle. We realized that this week, that increase was not enough to reach the goal. Do not give up. We are here together to achieve a more active life.

PAMN 06 - Hi, how are you? Changes are not so easy at first, but we know you can! We noticed his motivation to improve the quality of life, but we verified that this week this was not possible. How about starting in your building? The week's challenge is to climb at least once a day to the top floor by the stairs. Thank you for your participation.

PAMN 07- Hello. This week we were unable to reach the goal. When you start practicing physical activity, it is reasonable to have a lot of muscle pain and maybe generate dismay. Still, you are doing very well and increasing your muscle strength. Do not be discouraged.

PAMN 08 - Hello, we found this week that you could not beat your level of physical activity. Did you know that according to an IBGE survey of 2015, in Brazil, 52 million people have dogs? Are you one of them? If it is not, you certainly know a friend or neighbor who has a dog. How about increasing your daily physical activity. Take your dog for a walk on a more extensive course, or more than once a day? Alternatively, combine with a friend or neighbor to accompany them when you go for a walk with your dog. I'm sure it will be an excellent time to talk, and everyone involved will benefit from a cute little dog.

PAMN 09- Hello, have you done your physical activity today? We have seen this week that you have not reached the desired goal. Know that physical exercises do not change only your body; it changes your mind, your attitudes, and your mood. Come on! Good things happen to those who wait, but the best occur to those who get up and do it!

PAMN 10- How is it going? We are following his performance. We would like you to know that physical exercise brings various improvements such as increased muscle strength, improved bone density, reduced disease risks, chronic pain, and insomnia. We know there are weeks, like the last one, in which it is challenging to increase daily physical activity. You do not have to be the "first," or break records or win trophies, so little a professional athlete. You train to be your best. That is your purpose; you are your prize!

PAMN 11 - Hello. We have seen that we have not been able to reach our goal last week. Be more active on your commute to work! An excellent opportunity to become more productive is in shifting to your job. You can change the car or public transport by walking or cycling. For those who do not know how to pedal, there are significant 3-wheeled bikes. You can also park two or more blocks away from your work, or go down 1 point before and walk to your work. Changing today is always better than tomorrow!

PAMN 12 - Hi, how are you? We realized that even though you are an active person, you have not increased your number of steps in the past few days, we would like to help. Today is a great day to make a change. How about enjoying the beautiful weather that is out there and starting today? Taking a few more steps a day is associated with dramatically improves health and decreases the risk of developing cardiovascular disease.

**POSITIVE/ACHIEVED THE META - PCCP (pre-contemplative and contemplative POSITIVE)**

PCCP 01 - Hello, you are doing very well in the program. The objectives are being achieved. We will soon increase our challenges; we believe you are ready. Santos has landscapes that provide a great experience to perform exercises. How about enjoying some of them? Physical activity, even if in the small amount, already brings excellent health benefits.

PCCP 02 - You are doing a great job; we observe that you are taking more and more steps each day. These few more steps make a big difference in health. How about enjoying the weather today and taking a walk?

PCCP 03 - Hello, we believe that you look great as we see an increase in your daily physical activity. Obstacles along the way are being overcome day by day. A sedentary lifestyle is a risk factor for developing various diseases, and we are happy to see that you do not fit into this group. Keep it up, enjoy this day to increase your workouts even more.

PCCP 04 - Hi! That regular physical exercise practice brings innumerable benefits to people's bodies and minds are impossible to deny. To further assist in this task, you can count on your handsets and smartphones. Lately, many applications have come up to make life easier for those interested in practicing physical activity. That's why we're tracking your performance next to a mobile app and associated with our team; we'll be able to reach your goal. We trust you!

PCCP 05 - Hello! We are happy with your performance in our project. Some obstacles have already been overcome, and we want you to prepare for more. Every change has a positive effect on health. We want you to plan to try to increase your number of steps daily. We understand that it is difficult and that this process is complicated, but, according to your significant progress so far, we are confident that you will succeed.

PCCP 06 - Hello, how are you? We are happy with your participation in the project. We have already become familiar with this entire application process, and now we have new challenges. Doing physical exercise is very good for your health, but it can be a bit tiring in the beginning. It is normal to feel tired, and even some muscle aches the next day, but that is part of the adaptation. Let's focus on the reward, the health we will gain. Enjoy that Santos has beautiful landscapes, call a friend, and take a hike today. What about it?

PCCP 07 - Hi, how are you? You may have heard that physical exercise is good for your health, right? We also understand that in the beginning, they can be a bit difficult. We would like to offer you a challenge: How about enjoying today's pleasant weather and starting a hike today? To get better, we advise you to invite a friend or relative. Tiredness at the beginning is usual but focus on the benefits this will bring to your health. We trust you.

PCCP 08 - All right? How are you? We are pleased with your performance on our project. We understand that changing the habit of a healthier life is complicated, but we are here to help. Today is a pleasant climate to take a walk. Keep in mind that a few more steps make a big difference to our health. We suggest you invite someone and take a walk enjoying the afternoon in Santos, maybe take a peek at the sea. What do you think?

PCCP 09 - Hi, how are you feeling today? We observe that you are becoming more participatory in the project; your daily steps have increased, making us very happy. Its further steps generate significant benefits to your health, from decreasing body fat to protecting your heart. Keep it up, it's perfect, but it can get better. Enjoy this week of days that have no rain and plan some outdoor exercise sessions.

PCCP 10 Hello, how are you? We've been together for a couple of days, and we realize that you're already increasing your daily step numbers. Obstacles are gradually overcome; we have small goals for achieving them. Physical activity benefits are already apparent, such as reducing body fat and decreasing the risk of developing heart disease. As you are doing well, we want to increase the challenge. How about enjoying the day and taking a walk in the late afternoon? Call a friend from work or someone you have not seen for a long time to make the activity more enjoyable. Remember the benefits; we trust you.

PCCP 11 - Have you seen how the day is? We believe it is an excellent day for you to increase your physical activity a little. At first, it is more difficult, but with the day's passing, the results are appearing. Physical exercise makes us more willing to face day-to-day activities and contribute to the reduction of body fat and the prevention of heart disease. Enjoy today to start the change.

PCCP 12 Hello, how are you? We are pleased with your participation in our project. We have already seen promising results; your physical activity is increasing. It may seem like little, but those extra steps already contribute to improving your health. As we realize that you are responding well, we suggest that you increase slightly if some levels per day. Simple things like getting off the bus a point before, or climbing the stairs instead of the elevator will contribute even more. We believe you will remember the benefits you will receive by increasing these small attitudes in your daily life.

**POSITIVE/ATTACKED THE GOAL - PAMP (preparation, action and POSITIVE MAINTENANCE)**

PAMP 01 - Hi, we noticed that you had performed a lot of physical activity. That can generate some muscle discomfort, but do not get discouraged by it. Remember that this is the sign that your body is regenerating and preparing for the next challenge, so do not slow down. You are doing very well.

PAMP 02 - Hello, how are you? We are impressed with your performance in the project. Your data shows that you are changing your daily habits, and most importantly, to healthier habits. Physical activity generates numerous health benefits, such as reducing the risk of developing diseases and improving life quality. Undoubtedly you are already enjoying these benefits. Continue steadily on the project.

PAMP 03 - Hi, how are you? First, we would like to congratulate you on the evolution of the project. Your level of physical activity is increasing considerably. So we have a proposal, how about taking advantage of the pleasant weather today and running afterward? Or do some other sports? For those who have done so well so far, this challenge will be easy. We count on you.

PAMP 04 - So, how are you? You're feeling generous, we're watching your performance, and we see a significant evolution. Know that these steps that you have given more per day contribute a lot to improving your health. In addition to reducing body fat, your heart is more protected against the development of diseases. Our city is rich in beautiful landscapes. Enjoy this privilege and develop some outdoor activities. Call some friends, and your physical activity will become more fun.

PAMP 05 - Hi, how are you? We believe everything is fine. You were already on a reasonable level of physical activity, and now it's even better. This increase in physical activity at the beginning can generate some discomfort, such as muscle aches and even tiredness, but remember that these discomforts are signs of your body adapting, which is preparing for ever more significant challenges. So do not get discouraged. Keep your pace. You have to win.

PAMP 06 - Hello, how are you? We are delighted with your participation. For evolution to occur, the obstacles must become increasingly difficult, and consequently, the rewards will be higher. You already have considerable physical activity, but we believe you can do better. Try to adopt habits such as walking to nearby places or avoid using elevators. This increase will make a difference in its development, contributing to improving its health and quality of life.

PAMP 07 - A city with beaches and tourist landscapes like Santos is for few, right? We should take this opportunity to perform physical activity outdoors, which generates numerous health benefits, such as decreased body fat and stress. Invite some friends to practice some sport. In this phase, you are vital to keeping exercising, and diverse activities can be a good one.

PAMP 08 - Hello, how are you? We are impressed with your performance. His number of steps increased considerably. At this time, there may be some discomfort, such as muscle aches, but you must maintain the activity. These discomforts are signs that your body is adapting. Now that he's adjusting, we cannot stop, right? Enjoy the weather today and do some activity in the late afternoon or evening. Remember that you are gaining a lot in health with all of this.

PAMP 09 - Hello, we are starting our work with you with the conviction that we will achieve our goals. This week's difference between your body and next week is what you do in the next seven days to achieve your goals. Today is the perfect day to start our goals, further increasing the level of physical activity you already have. We trust you.

PAMP 10 - Hello, have you done your physical activity today? Being an active person, I imagine your answer to be yes. Did you know that the increase of 2000 steps per day reduces cardiovascular disease risk by about 10%, including in people with hypertension and diabetes? How about we start these changes today? These are attainable goals made for you, so we trust that you will achieve it.

PAMP 11- How are you? Nowadays, most people complain about lack of time, but simple changes in routine can increase their already considerable physical activity level. How about you change the elevator up to the stairs? It can be at work, at home, or the teaching location. If you are accustomed to going up the stairs in any of these, change at least once in the day the elevator and use the stairs, you will have significant benefits. We believe in your potential in improving your daily habits.

PAMP 12 - How are you? You are a person who knows that practicing physical activity is essential, but did you know that physical activity decreases the risk of cardiovascular diseases, such as the control and prevention of high blood pressure? Also, psychosocial benefits include improved self-esteem, reduced anxiety, stress, and social isolation, improving mental health in general. That's why we're here to help you get all these benefits. Continue to do physical activity! We believe in you.
